# Analysis of Skin Cancer and Patient Healthcare Using Data Mining Techniques

**DOI:** 10.1155/2022/2250275

**Published:** 2022-09-26

**Authors:** N. Arivazhagan, M. A. Mukunthan, D. Sundaranarayana, A. Shankar, S. Vinoth Kumar, R. Kesavan, Saravanan Chandrasekaran, M. Shyamala Devi, K. Maithili, U Barakkath Nisha, Tewodros Getinet Abebe

**Affiliations:** ^1^Department of Computational Intelligence, SRM Institute of Science and Technology, SRM Nagar, Kattankulathur 603203, India; ^2^School of Computing, Department of Computer Science and Engineering, Vel Tech Rangarajan Dr.Sagunthala R&D Institute of Science and Technology, Avadi, Chennai, India; ^3^Department of Computer Science and Engineering, Veltech Rangarajan Dr. Sagunthala R &D Institute of Science and Technology, Avadi, Chennai, India; ^4^Department of ECE, Manakula Vinayagar Institute of Technology, Puducherry, India; ^5^Institute of Computer Science and Engineering, Saveetha School of Engineering, Saveetha Institute of Medical and Technical Sciences, Chennai, India; ^6^Department of Computer Science and Engineering, SRM Institute of Science and Technology, Ramapuram, Chennai, India; ^7^Department of Information Technology, Sri Krishna College of Engineering and Technology, Coimbatore, India; ^8^Department of Chemical Engineering, College of Biological and Chemical Engineering, Addis Ababa, Ethiopia

## Abstract

Skin cancer is the uncontrolled growth of irregular cancer cells in the human-skin's outer layer. Skin cells commonly grow in an uneven pattern on exposed skin surfaces. The majority of melanomas, aside from this variety, form in areas that are rarely exposed to sunlight. Harmful sunlight, which results in a mutation in the DNA and irreparable DNA damage, is the primary cause of skin cancer. This demonstrates a close connection between skin cancer and molecular biology and genetics. Males and females both experience the same incidence rate. Avoiding revelation to ultraviolet (UV) emissions can lower the risk rate. This needed to be known about in order to be prevented from happening. To identify skin cancer, an improved image analysis technique was put forth in this work. The skin alterations are routinely monitored by this proposed skin cancer categorization approach. Therefore, early detection of suspicious skin changes can aid in the early discovery of skin cancer, increasing the likelihood of a favourable outcome. Due to the blessing of diagnostic technology and recent advancements in cancer treatment, the survival rate of patients with skin cancer has grown. The strategy for detecting skin cancer using image processing technologies is presented in this paper. The system receives the image of the skin lesion as an input and analyses it using cutting-edge image processing methods to determine whether skin cancer is present. The Lesion Image Analysis Tools use texture, size, and shape assessment for image segmentation and feature phases to check for various cancer criteria including asymmetries, borders, pigment, and diameter. The image is classified as Normal skin and a lesion caused by skin cancer using the derived feature parameters.

## 1. Introduction

The Skin cancer is the irregular enlargement and proliferation of skin-cells. It will be in different stages. All three types of dermatitis are very vulnerable [1]. Bone cancer, squamous cell carcinoma, melanoma, or carcinoma is all named after specific cells that cause them. Skin cancer is caused by the delicate epidermis of the skin so cancerous growths can be easily detected. Thus, unlike other cancers, it can be detected early and treated appropriately [2]. So the death toll from this type of cancer is lower than in other cancers. An Ultraviolet ray is the primary cause of skin cancer. Thus, the number of skin cancers is higher than other cancers [3]. Bone cancer can occur on the face. These are not contagious. It can be easily cured by surgery or radiation. Squamous cell carcinoma is somewhat migratory. But carcinoma is the rarest and most dangerous of all skin infections. This can have a serious impact as they are migratory [4]. These cancers can be detected by different symptoms. Suspicious symptoms include a change in the skin, sores on the skin, discoloration, or discoloration of an existing mole [5]. Therefore, even if the edges of the mole become irregular or the mole continues to enlarge, it is still considered a sign of cancer [6].

Skin cancer is one of the most common problems in the industry. Once this is detected, treatment is more likely to reduce it. Melanoma is the most serious type of skin cancer. However, it can be cured if detected early [7]. But this type of cancer can be dangerous if diagnosed too late. Melanoma is a tumor that develops from cells that pigment the skin. These cells, melanocytes, form a pigmented substance called melanin under the influence of ultraviolet radiation [8]. They are found in large numbers in the navy or mole. Degeneration of melanocytes occurs as a result of exposure to a number of factors: UV radiation, mechanical injury, heat or chemical burns, etc. Melanoma is more dangerous than all other types of skin cancer because it invades metastases and other organs quickly through the blood vessels [9]. Melanoma is very serious, but early detection can cure it. So, focus on the skin and especially the mole. Not everyone is at the same risk of developing melanoma. But if any of the following statements apply to you, be sure to check with your dermatologist regularly [10].

Melanoma is a skin cancer. It develops in the previous nevus (mole). It may be black or the skin color. When the nevus starts to grow large or ulcerative you know it is melanoma. Melanoma can cause metastases to other skin and nonskin organs [11]. Melanoma is a skin cancer. It develops in the previous nevus (mole). It may be black or the skin color. When the nevus starts to grow large or ulcerative you know it is melanoma [12]. Melanoma can cause metastases to other skin and nonskin organs. In most cases, melanoma develops on the face of the patient or on the body of the infected person [13]. Also in women, it usually develops in the lower legs, the most interesting thing about melanoma skin cancer is that it can occur in that part of the human body that is not exposed to the sun. Melanoma begins in cells that produce skin cancer pigment. These cells are called melanocytes [14–19]. Melanin is the result of these cells responsible for the color of the skin, eyes, and hair.

Most skin cancers are diagnosed and treated early, although malignant melanoma can be a bit difficult to treat effectively [21]. The mainly ordinary early symptom of skin cancer is an abnormal growth on the skin or a change in the color of the skin markers, small animals, and tags. Melanoma is often ulcerated and is prone to inflammation and bleeding. Some forms of skin cancer appear as incurable lesions or suspicious signs on the skin. If any of these symptoms occur, consult a doctor for a full examination and biopsy [22].

Further tests will be carried out to see if the melanoma has spread. If such a condition and melanoma becomes metastatic, additional treatment, such as chemotherapy, radiation therapy, or immunotherapy, may be needed, depending on how far the cancer has spread [23]. Similarly, squamous cell carcinoma is caused by sunlight and requires early treatment to prevent it. Other examples of treatment for skin cancer include laser surgery, cryosurgery, cure, and phototherapy. Regular checkups following skin cancer treatment are very important to make sure the cancer does not return. The major contributions of this work are as follows:

- Make it possible for researchers and practitioners to create deep learning models using image analysis technique
- To use deep learning to more accurately classify the cell images and identify skin cancer

The organization of the paper is as flows.  Section 2 explains the Literature survey.  Section 3 explains about Skin cancer and its types.  Section 4 explains the details about the proposed work.  Section 5 shows the results and discussion added to explain the results.  Section 6 concludes the research work with a summary.

## 2. Literature Review

Try to stay healthy and nutritious during treatment. Prepare meal-plans and take nutritional supplements to make sure your body is getting all the nutrients it needs. Follow the list of recipes you should eat during your cancer treatment [1]. Invest your time in various energy therapies and stretching exercises that will keep you healthy during therapy. It can do yoga to reduce the workload of cancer treatment. These activities will help you stay healthy [4]. Chemotherapy sessions and cancer treatment can affect anyone. It is important to ensure the emotional and spiritual well-being of the patient. Be clear with the various mind-body approaches that will help you stay calm [7]. Take care of your surroundings and make sure they prevent cancer. Avoid the use of personal care products, lamps, and cooking utensils that prevent carcinogenic compounds and other potential sources of radiation. This will make your home safer and reduce your chances of getting cancer [9]. Regular self-identification is the finest method to diagnose skin cancer in its premature period. It is important to check the human-skin regularly and if any abnormalities are found, consult a dermatologist for a thorough examination [10].

Over the years, tremendous progress has been made in the study of image-based skin cancer detection. Many various methods have been tested. By holding a challenge competition, the International Skin Imaging Collaboration (ISIC) event in 2018 became a de facto benchmark in the diagnosis of skin cancer. In addition, it has been stated that skin cancer can be found using a mobile app. Through all of these initiatives, researchers have worked to increase the diagnostic accuracy by using various categorization algorithms and approaches. With the development of convolutional neural networks (CNNs), image classification reached new heights. They classified images using CNNs. The most cutting-edge techniques for classifying images are CNNs, which essentially imitate the human visual cognitive system. Despite the abundance of work on picture classification, we focus only on deep learning techniques for photos of skin cancer in our overview of the literature. According to the ABCD rule of dermoscopy, of the four characteristics of asymmetry: border irregularity, color, and diameter, asymmetry is given the greatest prominence. Numerous studies on quantifying peer review have been conducted under the supervision of the scientific committee of the International Conference on Computer, Communication, and Convergence (ICCC 2015). Some methods determine the symmetry feature based on geometric measurements made of the entire lesion, such as the symmetric distance and circularity. As a metric for the irregularity of boundaries in dermoscopy images, other research suggests the circularity index [24–46].

The classification rate can be improved using the suggested approach. The extracted features increase the classification accuracy. The amount of the dataset also affects performance. The moment of execution is really important. The algorithms selected thus far for the suggested method are comparatively superior. In addition, a comparison of the employed classification methods yields an accuracy rate. And, from there, the best classification method can be refined and finalized.

## 3. Background of Skin Cancer

The Skin diseases in the human body are somewhat difficult to diagnose. It shows without feeling the signs of having skin cancer. Such diseases, which until now have been a problem for many, can be diagnosed and treated accordingly in the future. This has been made possible with the help of artificial intelligence technology. Artificial intelligence software has been developed that is capable of detecting the presence of skin disease in our body. This artificial intelligence software can accurately confirm the presence of skin disease in our body by comparing photos. Various photos and data have been used to diagnose skin cancer. Various photos of skin cancer were provided in order for the answers provided by the software to be valid.

These images were then tested at various stages. Artificial intelligence software has confirmed the accuracy of various skin cancers. The cancer-causing cell is named for different types of skin cancer. Cancer is also called carcinoma. Squamous cell carcinomas are often grouped together with basal cell carcinomas and are referred to as common skin cancers.

Melanoma: this is a very serious skin cancer. It tends to spread quickly. Melanoma preserves turn into serious in 6 weeks if not properly treated for skin cancer. If missing or unprocessed, melanoma preservation extends to an additional division of the human body. Melanoma can also appear on exposed skin. It has a random blurred exterior and is flat. Melanoma can be of more than one color. Most often, it develops on an existing mole on the skin. It can also appear as a black spot on the skin. Early diagnosis is important for the treatment of melanoma. Nodule growth is a very dangerous kind of tumor that should be treated as soon as possible. It looks different from other melanomas and has a uniform tone. This type of melanoma is known to grow fast. Therefore, the patient should be treated for skin cancer as soon as possible. Studies have shown that melanoma can be cured in the early stages.

Squamous-cell-carcinoma (SCC): it is the next the majority frequent type of skin cancer. Doctors use chemotherapy, radiation, or biological treatment to treat cancer. Cancer is not very easy to treat, it can take a lifetime, but only some simple precautions are needed to reduce the risk. Avoiding tanning beds is the first and most important factor. Another simple step is to wear sunscreens with an SPF (Sun Protection Factor) of over 15. It is best to wear hats, masks, and tightly woven clothing to block sunlight and UV rays. Lastly at 10 am and 4 pm stay away from sunlight when the sunlight is very strong and the amount of UV is high. SCC is more likely to develop on the face, neck, arms, ears, and back. Squamous cell carcinoma is present in approximately 20% of the total number of skin cancer patients. It is more common in people with immunodeficiency. Often, its behavior is similar to that of basal cell carcinoma. The only difference is the small chance of long-distance transmission. People with mild skin are more likely to develop SCC. Precancerous skin growth can also lead to SCC development. Occasionally, actinic Kerasotes (AK) can form on a person's skin. AK is a dry or scaly spot on the skin. This premature skin development can turn into squalors cell carcinoma. AK is not a skin cancer. But, if left untreated, AK can turn into skin cancer.

Basal cell carcinoma: this is the most dangerous type of skin cancer. However, it is the most common type of skin cancer. It grows slowly on the upper body, neck, and head. It can be dry and flaky or appear as a pearly lump. As it grows it can become sore and look like a sore that has not healed properly. Basal cell carcinoma, or PCC, is more likely to develop in people with fair skin. People with fair skin can also get this type of cancer. BCC develops after frequent exposure to the Sun for many years. It can also be caused by external tanning. PCC can form anywhere in the body. However, they are most common on the hands, head, and neck. Early skin cancer diagnosis and systemic cancer treatment are important in the case of BCC. If allowed, the BCC will grow deeper and penetrate the nerves and bones. This can lead to degeneration of the body.

Merkel Cell Cancer: it is one of the rare types of skin cancer. Researchers have concluded that almost everyone who develops Merkel cell carcinoma is over 50 years old. Although it is rare, Merkel cell carcinoma can be aggressive. It is advisable to seek treatment from a certified dermatologist or radiation oncologist. Patients diagnosed at an early stage of MCC are known to have the best outcomes in terms of cancer treatment. Other rare skin cancers are sebaceous carcinoma and coetaneous D-cell lymphoma.

**Figure 1:**
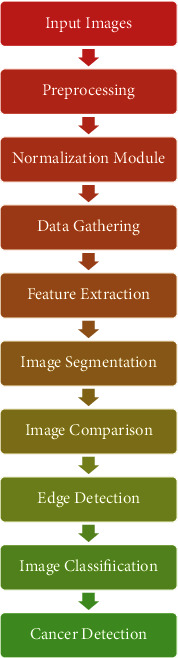
Proposed model block diagram.

The Skin cancer can develop primarily in the exposed areas of the skin such as the scalp, face, lips, ears, neck, chest, arms, hands, and legs in women. However, it can also occur in areas that are rarely exposed to sunlight. For example, this occurs below the palms, fingernails and toenails, and genitals. Skin cancer affects all skin tones. Changes in the skin are a warning sign for various skin cancers shown in Figure 1. Being alert to changes in your normal skin can help you get an earlier diagnosis. Some skin cancer symptoms are as following:

- Skin lesions are new moles, abnormal growths, scaly patches, bumps, sores, or blackheads that do not heal or disappear
- The two areas of asymmetric lesions are not identical
- Boundary lesions have ragged and irregular borders
- Color: these spots on the skin are unusual in color, such as white, red, pink, black, or blue
- The diameter is larger than the diameter of the space. The dot is larger than a quarter of an inch

## 4. Proposed Work

This research presents a method for identifying skin cancer using image processing tools. In order to identify whether skin cancer is present, the system inputs an image of the skin lesion and analyses it using cutting-edge image processing techniques. In order to check for several cancer criteria, such as asymmetry, borders, pigment, and diameter, the lesion image analysis tools use texture, size, and formative assessment for image segmentation and feature phases. Using the generated feature parameters, the image is categorised as Normal skin and a lesion caused by skin cancer.

A dermatologist will perform a skin test to get a definitive skin cancer diagnosis. In most cases, the appearance is enough to make a diagnosis and go to the treatment of skin cancer. Skin biopsy is used to confirm the development of skin cancer. Local anesthetics such as lidocaine are used to numb the area under the tumor and to perform a biopsy. After the anesthetic is applied, a small portion of the tumor is excised and sent to a pathologist for examination. The pathologist examines the tissue under a microscope and diagnoses skin cancer based on the characteristics of the extracted tumor. Skin cancer biopsy provides information on the stage of cancer. After considering these factors, cancer patients can be treated with one or a combination of these following skin cancer treatments:

- Cryotherapy: growth is frozen by the doctor in liquid nitrogen and destroyed when the tissue dissolves
- Surgical surgery: incision of the cancerous area along with the surrounding healthy skin
- Moss surgery: in this method, the cancerous growth is removed layer by layer and each layer is examined under a microscope until no cancerous cell is found
- Curettage and Electrification: this involves the removal of cancer cells using an elongated spoon-shaped blade and burning the remaining cancerous area with the help of an electric needle
- Chemotherapy: to kill cancer cells, drugs are taken orally, topically, or by injection or IV (intravenously) line
- Photodynamic therapy: a combination of laser light and certain drugs used to destroy cancer cells
- Radiation: high energy beams are used to destroy cancer cells
- Biological treatment: stimulates the immune system of the cancer patient to fight cancer cells using biological therapy
- Immunotherapy: to stimulate the cancer patient's immune system, a cream is applied to the skin of the cancer patient

To determine the severity of skin cancer, your doctor will determine the extent to which the cancer has spread to your lymph nodes or other parts of the body. There are 5 different levels of skin cancer (SC). (Algorithm 1)

SC-Level 0. This type of cancer does not go beyond the epidermis. It can be cured by removal or minor surgery. For melanoma on sensitive skin areas such as the face, some doctors rely on Moss surgery.

SC-Level 1. In this condition, the small tumor may spread to the second layer of skin, the dermis. Stage 1 melanoma is treated with a wide incision to remove the edges of the tumor and the normal skin around it. Some doctors prefer a sentinel lymph node biopsy (SLNB). If cancer cells are found in the lymph nodes, the doctor will carefully monitor the lymph nodes every few months with an ultrasound node. With this procedure, some immunoassay inhibitors or targeted medications are given.

SC-Level 2. These tumor cells do not spread from the original tumor, but may be larger and thicker, and may have other symptoms, such as scaling, bleeding, and shedding. The standard treatment for stage 2 melanoma is the extensive incision. SLNB is also performed in this condition because of the spread of cancer in the lymph nodes. Along with this targeted treatment, medications are also used.

SC-Level 3. In this condition, the cancer spreads or spreads to the lymph nodes and nearby skin and tissues. For the treatment of stage 3 melanoma, the primary tumor is surgically removed by separating the lymph node. After surgery, additional treatment, such as immunosuppressive inhibitors or targeted therapies (for cancer with BRAF GM), may be given to reduce the risk of recurrence of melanoma. Other options for treatment include the D-VEC vaccine, the Basil Calmet Gerin (BCG) vaccine, or the injection of interleukin-2 directly into melanoma. According to studies, the chances of survival are 63.6%.

SC-Level 4. This stage is the most advanced stage of melanoma. In this condition, the spread of the cancer goes beyond the original primary tumor to the lymph nodes, surrounding skin, tissues, and organs. This enlarged tumor is surgically removed and treated with radiation therapy, immunotherapy, and chemotherapy. The survival rate is only 22.5%.

### 4.1. Input Images

Here, we show how to classify skin lesions using an improved image analysis algorithm that is trained entirely from raw images with only the inputs of pixels and disease labels.

### 4.2. Preprocessing

Preprocessing is the initial stage of detection to enhance the quality of photographs, removing the undesired elements from the backdrop of the skin images and irrelevant noises.

### 4.3. Normalization Module

By using the normalisation technique, the variables in a particular sort of data can be changed to a specified scale without modifying the image's contrast or shape.

### 4.4. Data Gathering

The patient data can be divided into discrete categories or categorised in a dichotomous manner (if the feature is present: yes or no).

### 4.5. Feature Extraction

As a result of a comparison of the feature values extracted during the feature extraction stage, the skin lesion is categorised as either melanoma skin cancer, normal skin, or a mole. Think alphabetically, using the ABCDEs of melanoma, to recall prevalent traits of the disease. Asymmetry, border, color, diameter, and evolving are all abbreviations for ABCDE. The following aspects of skin damage are taken into account by doctors when diagnosing and classifying melanomas.

### 4.6. Image Segmentation

Image segmentation is carried out after preprocessing to extract the region of interest, in this case the skin lesion.

### 4.7. Image Comparison

Images utilizing the ABCD rule to compare benign and malignant skin lesions.

### 4.8. Edge Detection

In this paper, the edge identification of malignant skin lesions is the main focus. Edge detection algorithms have been utilized to identify the border of skin cancer lesions from their thermal pictures for the same purpose. While some algorithms failed to produce the desired outcomes, other methods showed promise in highlighting the lesion border, suggesting that they could be improved to produce better outcomes.

### 4.9. Image Classification

Malignant melanomas vs. benign nevi and keratinocyte carcinomas versus benign seborrheic keratosis were two binary classification problems that were taken into consideration. Both clinical and dermatoscopic pictures were used for the final categorization distinction.

### 4.10. Cancer Detection

Models that help in improving the accuracy of skin cancer prediction are built using a model-driven architecture that implements deep learning techniques at the core.

## 5. Results and Discussion

The proposed Enhanced image analysis technique (EIAT) was compared with the existing Multiclass skin diseases classification (MCSDC), Decision support system for detection and classification (DSSDC), Automatic skin cancer detection (ASCD) and Skin lesion classification using convolution neural network (SLCNN).  Table 1 through 5 express the comparison of the proposed and existing methods in terms of accuracy.

### 5.1. Early Skin Detection

The prognosis of skin cancer is, in general, better than that of some other cancers. These cancers can be found as part of the cause, so they are diagnosed in earlier, more curable stages. This means that frequent testing of your skin is important. While doctors have a trained eye, not everyone can see a dermatologist regularly and no one is motivated to take care of your health. Since all races, skin colors, and adults can get skin cancer, it is a good idea for everyone to have their skin tested on a regular basis. The guidelines for doing self-skin tests vary by system. The following Table 1 expresses the early detection comparison between the existing and proposed methods.

### 5.2. Skin Cancer Screening

In addition to physical examination, skin cancer screening usually includes education about skin cancer and its risk factors. The doctor will explain how to deal with sunlight and give tips on how to protect yourself from skin cancer and contraindications found: if exposed skin areas are found during the skin cancer test, the treating physician may take a sample of the tissue, which is then sent. The tissue sample is then prepared and cut so that it can be evaluated under a microscope. The following Table 2 expresses a skin cancer screening comparison between the existing and proposed methods.

### 5.3. Skin Changes Monitoring

Everyone should regularly check their body for suspected skin changes. Use a well-lit room or daylight for this purpose, as it is the only way to get an optimal view of skin changes. Do not forget to check under your toes and feet for abnormalities. Ask someone close to you to check the back and parts of the body that are hard to see. Almost everyone has a mole on their body. The term skin cancer refers to a variety of malignant diseases of the skin. The initial stage can vary considerably depending on the specific disease and the type of degenerated cell. The following Table 3 expresses the early detection comparison between the existing and proposed methods.

### 5.4. Black and White Skin Cancer Identification

There should be a difference between black and white skin cancers. There are many factors to take into account and they can help in the early stages of skin cancer. If it is asymmetrical, pale, and very large (more than 5 mm in diameter), has different colors and has changed over the last three months, a pigmented skin area will always be clear. Even if a pigmented skin area begins to itch, the skin should be carefully examined. The so-called white skin cancer usually develops at an advanced age and in areas exposed to ultraviolet light (for example on the face or hands). In the early stages, hardening of the skin is often seen in the affected area. The following Table 4 expresses the detection of black and white cancer cells comparison between the existing and proposed methods.

### 5.5. UV Protection

While Sun protection is an important part when using sunscreen, there is a difference that there are many things you can do. Not only has sunscreen increased the risk of skin cancer since it became available, but some dermatologists recommend sunscreen 10 minutes or 15 minutes before applying sunscreen. In addition, there is no evidence that sunscreen reduces the risk of melanoma, the type of skin cancer that causes the majority of deaths from the disease; however, it is still important to use sunscreen. The following Table 5 expresses the early detection comparison between the existing and proposed methods.

## 6. Conclusion

The chances of treating skin cancer with an early diagnosis increase dramatically. Once it is established that there is skin cancer, there are several treatment options. A surgical removal is often the first step, removing the melanoma and surrounding skin parts. Nonmelanoma skin cancer is the most common skin cancer in the world. Millions of people worldwide are diagnosed with this cancer each year. Clinical trials have shown that arytenoids can help heal ulcers caused by the Sun's radiation. After eating them, antioxidants, especially lycopene, clog the skin and prevent the formation of cancerous growths. The proposed Enhanced image analysis technique (EIAT) was compared with the existing Multiclass skin diseases classification (MCSDC), Decision support system for detection and classification (DSSDC), Automatic skin cancer detection (ASCD), and Skin lesion classification using convolution neural network (SLCNN). The proposed model complications of skin cancer, such as metastasis, can also lead to symptoms. Skin cancers most often occur in areas exposed to the Sun but can occur anywhere. Common and uncommon symptoms of skin cancer can be found, as well as specific software that can signal a melanoma. Deep learning was applied in the paper's enhanced picture analysis technique. The tool's construction of a Deep Learning Model was illustrated in the publication that revealed its functionality. The procedure for gathering data and testing it against the suggested model to find cancer cells was described in the publication. The proposed model successfully identified cancer cells from images of cancer cells with an accuracy of 94.58% in early skin detection, 94.58% in skin cancer screening, 90.22% in skin changes monitoring, 85.49% in 5.4 Black and white skin cancer identification, and 90.07% in UV Protection comparison. Reference [20].

## Figures and Tables

**Algorithm 1:**
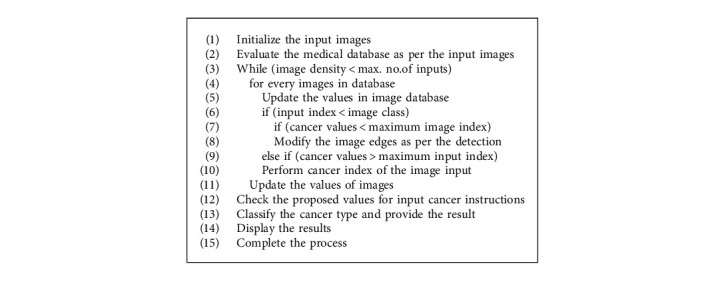
Enhanced image analysis technique (EIAT).

**Table 1:** Early detection comparison.

No of inputs	MCSDC	DSSDC	ASCD	SLCNN	EIAT
100	91.32	79.10	91.73	77.47	95.09
200	90.03	78.35	87.11	74.07	94.99
300	90.28	78.38	87.11	74.43	94.92
400	89.50	77.89	84.03	72.28	94.83
500	88.98	77.53	81.72	70.76	94.75
600	88.46	77.17	79.41	69.24	94.66
700	87.94	76.81	77.10	67.72	94.58

**Table 2:** Skin cancer screening comparison.

No of inputs	MCSDC	DSSDC	ASCD	SLCNN	EIAT
100	90.41	79.20	87.58	75.62	94.88
200	90.33	79.29	87.78	75.49	94.84
300	90.34	79.42	88.04	75.47	94.81
400	90.69	79.84	88.67	75.90	94.79
500	88.98	77.53	81.72	70.76	94.75
600	88.46	77.17	79.41	69.24	94.66
700	87.94	76.81	77.10	67.72	94.58

**Table 3:** Skin changes monitoring comparison.

No of inputs	MCSDC	DSSDC	ASCD	SLCNN	EIAT
100	79.03	83.51	84.61	68.25	97.49
200	77.76	82.91	82.89	67.25	95.85
300	76.50	81.36	82.59	66.52	95.12
400	75.23	80.44	81.34	65.61	93.78
500	73.97	79.37	80.33	64.75	92.60
600	72.70	78.29	79.32	63.88	91.41
700	71.43	77.21	78.31	63.02	90.22

**Table 4:** Detection of black and white cancer cells comparison.

No of inputs	MCSDC	DSSDC	ASCD	SLCNN	EIAT
100	73.97	79.37	80.33	64.74	92.60
200	72.70	78.29	79.32	63.88	91.41
300	71.44	77.22	78.31	63.01	90.23
400	70.17	76.14	77.30	62.15	89.04
500	68.91	75.07	76.29	61.28	87.86
600	67.64	73.99	75.28	60.42	86.67
700	66.38	72.92	74.27	59.55	85.49

**Table 5:** UV Protection comparison.

No of inputs	MCSDC	DSSDC	ASCD	SLCNN	EIAT
100	80.29	86.14	85.76	70.22	99.37
200	79.95	84.73	85.02	69.35	97.82
300	79.61	83.32	84.28	68.48	96.27
400	79.27	81.91	83.54	67.61	94.72
500	78.93	80.50	82.80	66.74	93.17
600	78.59	79.09	82.06	65.87	91.62
700	78.25	77.68	81.32	65.00	90.07

## Data Availability

The datasets used and/or analyzed during the current study are available from the corresponding author on reasonable request.
